# Effects of histidine and vitamin C on isoproterenol-induced acute myocardial infarction in rats

**Published:** 2016-03-15

**Authors:** Masoumeh Moradi-Arzeloo, Amir Abbas Farshid, Esmaeal Tamaddonfard, Siamak Asri-Rezaei

**Affiliations:** 1 *Postgraduate student, Department of Pathobiology, Faculty of Veterinary Medicine, Urmia University, Urmia, Iran; *; 2 *Department of Pathobiology, Faculty of Veterinary Medicine, Urmia University, Urmia, Iran; *; 3 *Department of Basic Sciences, Faculty of Veterinary Medicine, Urmia University, Urmia, Iran; *; 4 *Department of Internal Medicine and Clinical Pathology, Faculty of Veterinary Medicine, Urmia University, Urmia, Iran.*

**Keywords:** Histidine, Isoproterenol, Myocardial infarction, Propranolol, Vitamin C

## Abstract

In the present study, we investigated the effects of histidine and vitamin C (alone or in combination) treatments against isoproterenol (a β-adrenergic receptor agonist)-induced acute myocardial infarction in rats. We used propranolol (a β-adrenergic receptor blocker) to compare the results. Rats were given intraperitoneal injections of histidine (40 mg kg^-1^) and vitamin C (40 mg kg^-1^) alone and combined daily for 21 days. Propranolol (10 mg kg^-1^) was orally administered daily for 10 days (from day 11 to day 21). Myocardial infarction was induced by subcutaneous injections of 150 mg kg^-1^ of isoproterenol at an interval of 24 hr on days 20 and 21. Blood and tissue samples were taken for histopathological and biochemical evaluations following electrocardiography recording on day 21. Isoproterenol elevated ST segment, increased heart weight, heart rate, serum activities of aspartate transaminase, lactate dehydrogenase, creatine kinase-MB and heart tissue content of malondialdehyde, and decreased R wave amplitude and superoxide dismutase and catalase activities of heart tissue. Necrosis, edema and inflammatory cells infiltration were observed in myocardial tissue sections. Our results indicated that histidine and vitamin C alone, and especially in combination prevent isoproterenol-induced cardiotoxicity and have similar protective effects with propranolol. Cardioprotective effects of histidine and vitamin C may be associated with their ability to reduce free radical-induced toxic effects.

## Introduction

Acute myocardial infarction is a leading cause of morbidity and mortality worldwide.^1^ It occurs due to coronary artery blockage usually caused by atherosclerotic clot or spasm of the arteries.^2^^,^^3^ Coronary artery occlusion interrupts the coronary blood supply needed to satisfy myocardial demands, leading to oxygen and nutrient deprivation of the heart, eventually destroying cardiac tissue.^4^ Oxidative stress and inflammation play central roles in pathophysiology of myocardial infarction.^5^ Isoproterenol, as a synthetic β-adrenergic receptor agonist, at a supra- maximal dose produces myocardial infarction through production of highly cytotoxic free radicals.^6^ The rat model of isoproterenol-induced myocardial infarction offers a reliable non-invasive technique for studying the effects of various potentially cardioprotective agents.^7^ Propranolol, a β-adrenergic receptor antagonist, has been frequently used for its protective effect in isoproterenol-induced myo-cardial infarction in rats.^8^^,^^9^

Histidine is a semi-essential amino acid and has many biological functions such as anti-inflammatory, tissue protective and antioxidant properties.^10^^-^^14^ Although there are no reports showing the protective effect of histidine on isoproterenol-induced myocardial infarction, Obata *et al*.^15^ reported an inhibitory effect of histidine on iron (II)-induced hydroxyl radical generation in rat hearts. In this context, Farshid *et al*. reported a cardioprotective effect of histidine in doxorubicin-induced cardiomyopathy in rats. ^13^ Vitamin C is an important water-soluble antioxidant and enzyme cofactor in plants and animals.^16^ This vitamin exerts beneficial effects on cardiovascular system such as anti-arrhythmic, anti-atherosclerotic effects.^17^^,^^18^ It has been shown that vitamin C alone and in combination with other agents such as ferulic acid prevented isoproterenol-induced myocardial infarction.^19^^,^^20^

Since multiple factors such as inflammation, β-adrenergic system and oxidative stress are involved in the pathogenesis of myocardial infarction,^5^^,^^21^^,^^22^ this study was aimed to investigate the effects of separate and combined treatments with histidine and vitamin C on isoproterenol-induced myocardial infarction. We used propranolol to compare the results. Variables chosen to assess myocardial damage and protective effects of test agents included heart weight, electrocardiographic findings, serum activities of aspartate transaminase (AST), lactate dehydrogenase (LDH) and creatine kinase-MB (CK-MB), cardiac tissue malondialdehyde (MDA) content, superoxide dismutase (SOD) and catalase activities and histopathological evaluation of heart tissue.

## Materials and Methods

**Animals. **In the present study, 36 male Wistar rats weighed 250 to 290 g were used. They were housed in ventilated room at a temperature of 22 ± 0.5 ˚C with a 12 hr light/dark cycle. They were freely provided with food and water. The Veterinary Ethics Committee of Faculty of Veterinary Medicine of Urmia University approved the research and animal care procedures used in this study.

**Drugs and Chemicals. **Isoproterenol hydrochloride, histidine dihydrochloride, vitamin C (ascorbic acid) and propranolol hydrochloride were purchased from Sigma-Aldrich Chemical Co. (St. Louis, USA). All the analytical chemicals were purchased from Merck Chemical Co. (Darmstadt, Germany).

**Experimental groups. **In this study, 36 rats were divided into six groups as follows: Normal saline group was treated with intraperitoneal (IP) injections of normal saline for 21 days. In isoproterenol group, subcutaneous (SC) injections of isoproterenol (150 mg kg^-1^) were performed on days 20 and 21. Histidine group was treated with IP injections of histidine (40 mg kg^-1^) for 21 days. Vitamin C group was treated with IP injections of vitamin C (40 mg kg^-1^) for 21 days. In histidine plus vitamin C group, IP injections of histidine (40 mg kg^-1^) plus vitamin C (40 mg kg^-1^) were performed for 21 days. Propranolol group was treated with oral (PO) administration of propranolol (10 mg kg^-1^) for 10 days (from day 11 to day 21). In the present study, time schedules and dosages of histidine, vitamin C and propranolol were designed according to previous studies.^8^^,^^13^^,^^14^^,^^19^

**Induction of acute myocardial infarction. **For induction of acute myocardial infarction, isoproterenol was dissolved in normal saline and injected (150 mg kg^-1^, SC) on days 20 and 21 at an interval of 24 hr.^23^ Animals were euthanized 24 hr after the second dose of isoproterenol.

**Electrocardiography. **Electrocardiography (ECG) was recorded 24 hr after the second injection of normal saline and isoproterenol using ECG apparatus (Siemens, Forchheim, Germany). The rats were anaesthetized with IP injection of ketamine (80 mg kg^-1^) and xylazine (8 mg kg^-1^). In all animals, 15 min after anesthesia needle electrodes were inserted under skin for standard limb lead II recording at a paper speed of 50 mm sec^-1^ and 20 mm = 2 mV calibration. The ECG apparatus was calibrated at 1 mV/2 cm with speed of 50 mm sec^-1^. Five min later the ECG was recorded for five sec. Heart rate, R-R and QT intervals, R wave amplitude and ST segment were calculated from ECG recordings.^13^ The above-mentioned ECG parameters have been used in previous studies on isoproterenol-induced acute myocardial infarction.^23^^,^^24^

**Biochemical assay. **After ECG recording of anesthetized rats, a 23 gauge needle was inserted into the heart through 7^th^ and 8^th^ intercostal spaces. Blood samples were collected from the heart in tubes contain no heparin.^13^ These tubes were centrifuged at 3500 rpm for 10 min, serum samples separated and transferred to Eppendorf tubes for AST, LDH and CK-MB biochemical analyses. Immediately after blood sampling, the thoracic cavity was opened and the heart was removed, washed with normal saline and blotted dry on filter papers and weighed. Heart weight/body weight ratio was calculated according the following formula: 

$\mathrm{Ratio}(\%)=\frac{\mathrm{Heart weight}(g)}{\mathrm{Body weight}(g)}\times 100$

One half of the heart washed to prepare homogenates for biochemical analyses, and the remaining half was fixed in 10% buffered formalin for histological studies.^13^

Serum activities of AST, LDH and CK-MB were measured spectrophotometrically (LKB, Vienna, Austria) using their commercial kits (Man Co., Tehran, Iran for AST and LDH and Pars Azmoon Co., Tehran, Iran for CK-MB). Serum AST, LDH and CK-MB activities were expressed as units per liter.

Rat heart tissue was homogenized at 4 ˚C in 50 mM ice-cold phosphate buffer containing 1 mM EDTA (pH 7.4) to give 10% homogenate (w/v), using Polytron homogenizer (GlenMills Inc., Clifton, USA) for 5 min, followed by a sonic homogenizer for 3 min. The homogenates were then centrifuged at 1500 rpm for 15 min at 4 ˚C. The resulting supernatants were collected and stored at 0 ˚C before use. The MDA levels in the heart tissue homogenates were determined by thiobarbituric acid (TBA) method.^25^ A volume of 0.2 mL homogenate was pipetted into a test tube, followed by the addition of 0.2 mL of 8.1% sodium dodecyl sulfate (SDS), 1.5 mL of 30% acetic acid (pH 3.5) and 1.5 mL of 0.8% TBA. Tubes were boiled for 60 min at 95˚C and then were cooled on ice. Distilled water (0.1 mL) and 5.0 mL of n-butanol: pyridine (15:1 v/v) mixture were added to the tubes and centrifuged at 1500 rpm for 10 min. The absorbance of the developed color in organic layer was measured at 532 nm. Levels of MDA were expressed as nmol g^-1^ tissue. The SOD activity was determined in the supernatant using the nitro blue tetrazolium by method of Delides *et al*.^26^ This method employs xanhine and xanthine oxidase to generate superoxide radicals which react with 2-(4-iodo-phenyl)-3-(4-nitrophenole)-5-phenyl-tetrazolium chloride (INT) to form a red formazan dye. The SOD activity is then measured by the degree of inhibition of this reaction. One unit of SOD is that which causes a 50% inhibition of the rate of reduction of INT under the conditions of the assay. The activity of catalase was determined in 3 mL of reaction media, which contained 2 mL of homogenizing medium (phosphate buffer; pH 7.0) in a test tube followed by 1 mL of H_2_O_2_ solution.^27^ The blank was composed of one mL buffer pH 7.0 and 2 mL tissue homogenate (pH 7.0). The extinction was measured at a wavelength of 240 nm using ultraviolet-visible spectro-photometer (Camspec M330, Cambridge, UK). SOD and catalase activities were expressed as U mg^-1^ protein. Protein content in the heart tissue homogenate was estimated by the method of Lowry *et al*.^28^

**Histopathology evaluation. **The fixed heart tissues were processed routinely for paraffin embedding. For each sample, 4-5 µm thick sections were cut and stained by hematoxylin and eosin (H & E), and examined under a light microscope. Three sections were provided from each cardiac tissue. The evaluation of the heart sections was based on the severity of the pathological changes including confluent necrosis, hemorrhages, edema and inflammatory cell infiltration. The following scores were given to lesions observed: 0 – none, 1 – mild, 2 – moderate and 3 – severe.^13^

**Statistical analysis. **Statistical comparisons were performed using the GraphPad Prism (Version 5.0; GraphPad software Inc., San Diego, USA). Data obtained from normal saline and isoproterenol groups were analyzed using unpaired *t*-test. Differences among isoproterenol and treatment groups were analyzed by one-way analysis of variance (ANOVA) followed by Tukeyʼs test. Semi quantitative data analyses of histopathology subjected to Kruskal Wallis followed by Mann-Whitney test. Values were reported as mean ± SEM. The significance level was expressed as *p *< 0.05.

## Results

No significant differences were observed among treated groups with regard to body weight. Isoproterenol significantly (*p *< 0.001) increased heart weight and heart weight/body weight ratio. Alone (*p *< 0.05) and combined (*p *< 0.01) treatments with histidine and vitamin C significantly showed inhibitory effects on heart weight and heart weight/body weight ratio. These effects were similar to that of propranolol (Table 1).

**Table 1:** Effects of histidine (40 mg kg^-1^), vitamin C (40 mg kg^-1^) and propranolol (10 mg kg^-1^) on heart weight, body weight and heart weight/body weight ratio changes induced by isoproterenol (ISO; 150 mg kg^-1^) in rats. Data are presented as mean ± SEM

**Groups**	**Heart weight** ** (g)**	**Body weight** ** (g)**	**Heart weight/Body weight ratio** ** (%)**
**Normal saline **	0.654 ± 0.027	257.200 ± 9.900	0.255 ± 0.009
**ISO**	0.929 ± 0.050*	233.300 ± 7.100	0.402 ± 0.023*
**Histidine + ISO **	0.755 ± 0.033**	247.200 ± 8.700	0.307 ± 0.018**
**Vitamin C + ISO **	0.768 ± 0.031**	252.800 ± 6.600	0.304 ± 0.011**
**Histidine + Vitamin C + ISO**	0.727 ± 0.029***	261.300 ± 6.600	0.278 ± 0.009***
**Propranolol + ISO**	0.725 ± 0.034***	257.008 ± 7.700	0.281 ± 0.010***

* indicates significant difference compared to normal saline treated group at* p* < 0.001.

** and *** indicate significant differences compared to isoproterenol treated group at* p *< 0.05 and *p* < 0.01, respectively.

**Fig. 1:**
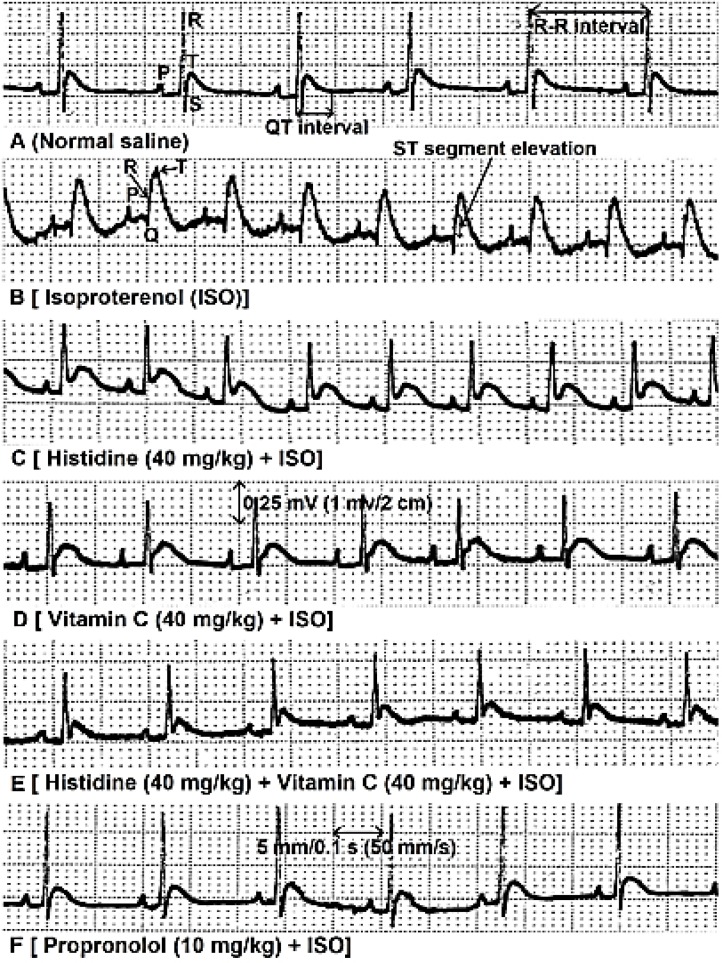
ECG recordings from control, isoproterenol (ISO) and histidine and vitamin C (alone and in combination) and propranolol treatments. Normal ECG recordings (**A**). Isoproterenol (**B**) produced cleared changes in R-R interval and R wave amplitude and ST segment. Histidine (**C**), vitamin C (**D**), histidine + vitamin C (**E**) and propranolol (**F**) prevented ECG changes induced by isoproterenol. Speed: 50 mms^-1^, Amplitude: 1 mV/2 cm

The ECG patterns of normal and experimental rats are shown in Fig. 1 and Table 2. Normal saline treated rats showed normal ECG pattern, whereas isoproterenol, with no significant effect on QT interval, significantly increased heart rate (*p *< 0.001), elevated ST segment (*p *< 0.0001) and R wave amplitude (*p *< 0.001). Separate and combined treatments with histidine and vitamin C significantly (*p *< 0.01) inhibited isoproterenol effects on ECG parameters. No significant differences were observed among propranolol and separate and combined treatments of histidine and vitamin C in inhibition of EGC changes induced by isoproterenol (Table 2).

**Table 2:** Effects of histidine (40 mg kg^-1^), vitamin C (40 mg kg^-1^) and propranolol (10 mg kg^-1^) on heart rate, R-R and QT intervals and R wave and ST segment amplitude changes induced by isoproterenol (ISO; 150 mg kg^-1^) in rats. Data are presented as mean ± SEM

**Groups**	**Heart rate ** **(bpm)**	**R-R interval ** **(sec)**	**Q-T interval ** **(sec)**	**R wave amplitude (mV)**	**ST segment amplitude (mV)**
**Normal saline **	268.300 ± 10.800	0.230 ± 0.011	0.060 ± 0.006	0.650 ± 0.050	0.027 ± 0.008
**ISO**	365.000 ± 12.000*	0.160 ± 0.006*	0.062 ± 0.005	0.330 ± 0.020*	0.180 ± 0.020**
**Histidine + ISO **	306.000 ± 15.800***	0.200 ± 0.009***	0.068 ± 0.009	0.520 ± 0.030***	0.085 ± 0.013***
**Vitamin C + ISO **	283.300 ± 12.300***	0.210 ± 0.009***	0.065 ± 0.006	0.540 ± 0.050***	0.078 ± 0.009***
**Histidine + Vitamin C + ISO**	273.000 ± 11.500***	0.220 ± 0.009***	0.068 ± 0.006	0.580 ± 0.050***	0.073 ± 0.011***
**Propranolol + ISO**	270.000 ± 10.600***	0.220 ± 0.010***	0.070 ± 0.007	0.620 ± 0.070***	0.040 ± 0.008†

* and ** indicate significant differences compared to normal saline treated group at *p* < 0.001 and *p* < 0.0001, respectively.

*** and † indicate significant differences compared to isoproterenol treated group at *p* < 0.01 and *p* < 0.001, respectively.

Isoproterenol significantly (*p* < 0.0001) increased serum activities of AST, LDH and CK-MB. Separate (*p* < 0.01) and combined (*p* < 0.001) treatments with histidine and vitamin C and propranolol significantly inhibited isoproterenol-induced increase of serum AST, LDH and CK-MB activities. Inhibitory effect of combined treatment with histidine and vitamin C was more significant (*p* < 0.01) than separate treatments of them and were similar to propranolol effects (Table 3). 

**Table 3:** Effects of histidine (40 mg kg^-1^), vitamin C (40 mg kg^-1^) and propranolol (10 mg kg^-1^) on serum level changes of aspartate transaminase, lactate dehydrogenase and creatine kinase-MB induced by isoproterenol (ISO; 150 mg kg^-1^) in rats. Data are presented as mean ± SEM

**Groups**	**Aspartate transaminase (U L** ^-1^ **)**	**Lactate dehydrogenase ** **(U L** ^-1^ **)**	**Creatine kinase-MB (U L** ^-1^ **)**
**Normal saline **	221.900 ± 12.700	91.700 ± 7.700	81.900 ± 7.000
**ISO**	566.600 ± 19.900*	235.900 ± 14.500*	266.600 ± 18.300*
**Histidine + ISO **	485.600 ± 15.700**	190.600 ± 10.700**	183.400 ± 10.200**
**Vitamin C + ISO **	434.500 ± 12.500**	171.200 ± 7.600**	176.400 ± 9.800**
**Histidine + Vitamin C + ISO**	315.100 ± 13.100***†	126.700 ± 9.400***†	125.100 ± 11.400***†
**Propranolol + ISO**	354.200 ± 13.400***	157.400 ± 10.100***	147.500 ± 11.900***

* indicates significant differences compared to normal saline treated group at *p* < 0.0001.

** and *** indicate significant differences compared to isoproterenol treated group at* p* < 0.01 and *p *< 0.001, respectively.

Isoproterenol significantly (*p* < 0.0001) increased MDA content and decreased SOD and catalase activities of heart tissue (Table 4). Histidine and vitamin C alone (*p* < 0.01) and in combination (*p* < 0.001) and propranolol (*p* < 0.01) treatments significantly inhibited isoproterenol- induced changes in heart tissue level of MDA and SOD and catalase activities. Inhibitory effect of combined treatment with histidine and vitamin C was more significant (*p *< 0.01) than separate treatments and also propranolol treatment (Table 4). 

**Table 4:** Effects of histidine (40 mg kg^-1^), vitamin C (40 mg kg^-1^) and propranolol (10 mg kg^-1^) on cardiac tissue malondialdehyde (MDA) content, superoxide dismutase (SOD) and catalase induced by isoproterenol (ISO; 150 mg kg^-1^) in rats. Data are presented as mean ± SEM

**Groups**	**MDA (nmol g** ^-1^ ** tissue)**	**SOD (U mg** ^-1^ ** protein)**	**Catalase (U mg** ^-1^ ** protein)**
**Normal saline **	4.100 ± 0.400	22.400 ± 1.600	31.900 ± 2.100
**ISO**	15.900 ± 0.700*	5.900 ± 0.700*	9.600 ± 1.000*
**Histidine + ISO **	9.400 ± 0.800**	13.100 ± 0.900**	15.900 ± 1.100**
**Vitamin C + ISO **	8.300 ± 0.600**	14.100 ± 1.100**	16.100 ± 0.900**
**Histidine + Vitamin C + ISO**	4.900 ± 0.800***†	21.800 ± 1.100***†	25.200 ± 1.100***†
**Propranolol + ISO**	9.200 ± 0.800**	15.800 ± 0.900**	17.500 ± 1.200**

* indicates significant differences compared to normal saline treated group at *p* < 0.0001.

** and *** indicate significant differences compared to isoproterenol treated group at* p* < 0.01 and *p *< 0.001, respectively.

† indicates significant difference compared to histidine and vitamin C used alone and propranolol at *p* < 0.01.

**Fig. 2.**
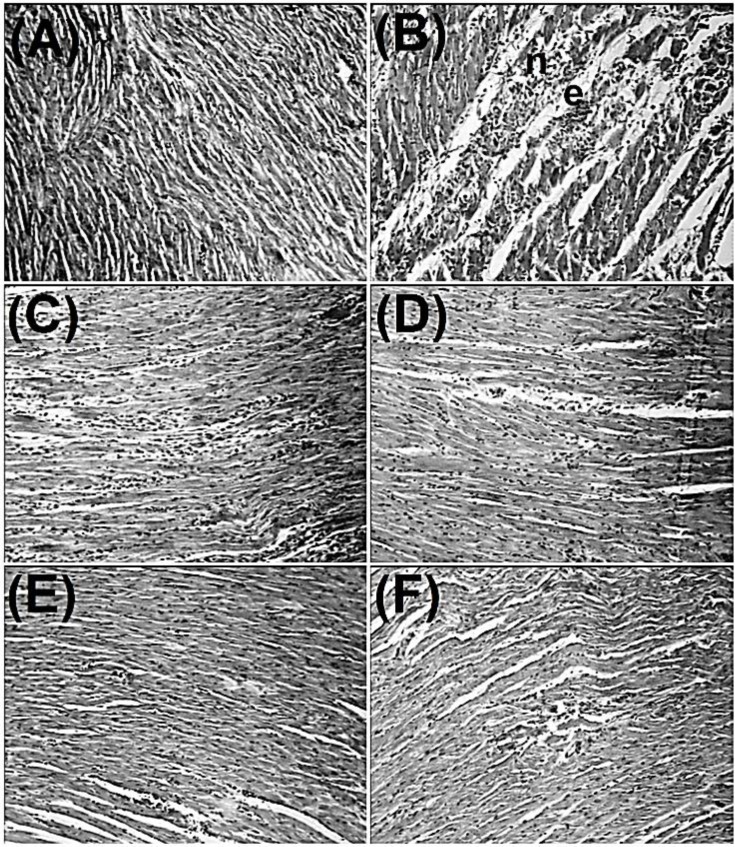
Effects of histidine and vitamin C (alone and in combination) and propranolol on isoproterenol-induced histopathological alternations in cardiac tissues. **A)** Normal cardiomyocytes; **B)** Confluent necrosis (n) and edema (e) are seen in isoproterenol treated group; **C** and **D)** Partial reduction of diffuse confluent necrosis and edema are seen in histidine (40 mg kg^-1^) and vitamin C (40 mg kg^-1^) treated groups, respectively; **E)** Moderate confluent necrosis and edema are seen in histidine (40 mg kg^-1^) plus  vitamin C (40 mg kg^-1^) treated rats; **F)** Minimal confluent necrosis and edema are seen in propranolol (10 mg kg^-1^) treated group (H & E, 100×).

Isoproterenol produced confluent necrosis, hemorrhages, edema and inflammatory cell infiltration in the heart tissue when compared (*p* < 0.0001) with normal saline treated group. Alone treatments with histidine and vitamin C and propranolol significantly (*p* < 0.05) inhibited isoproterenol-induced histopathological changes in the heart tissue. In addition, isoproternol-induced heart tissue changes were significantly (*p *< 0.01) inhibited with combination treatments of histidine and vitamin C (Fig. 2 and Table 5). 

**Table 5:** Effects of histidine (40 mg kg^-1^), vitamin C (40 mg kg^-1^) and propranolol (10 mg kg^-1^) on necrosis, hemorrhages, edema and inflammatory cell infiltration induced by isoproterenol in rats. Data are presented as mean ± SEM

**Groups**	**Confluent necrosis**	**Hemorrhages**	**Edema**	**Inflammatory cell infiltration**
**Normal saline **	-	-	-	-
**ISO**	2.830 ± 0.170*	2.670 ± 0.210*	2.670 ± 0.210*	2.500 ± 0.220*
**Histidine + ISO **	1.830 ± 0.170**	1.670 ± 0.210**	1.830 ± 0.170**	1.500 ± 0.220**
**Vitamin C + ISO **	1.670 ± 0.150**	1.500 ± 0.170**	1.500 ± 0.130**	1.330 ± 0.180**
**Histidine + Vitamin C + ISO**	1.170 ± 0.170**	1.500 ± 0.150**	1.500 ± 0.170**	0.830 ± 0.180**
**Propranolol + ISO**	1.330 ± 0.210**	1.170 ± 0.170**	1.330 ± 0.210**	1.500 ± 0.260**

* indicates significant differences compared to normal saline treated group at *p* < 0.001.

** indicates significant differences compared to isoproterenol treated group at *p* < 0.01.

## Discussion

Isoproterenol, as a synthetic catecholamine, acts on both β_1_, and β_2_-adrenergic receptors to produce myocardial infarction by using several mechanisms. Increase in heart weight might be associated with the increased water content, edematous intramuscular space, increased protein content and infiltration of inflammatory cells to damaged areas.^23^ The ECG is a good tool to analyze the function of the heart in physiological and pathological states.^29^ Increase of heart rate may be due to positive chronotropic effects of isoproterenol and inadequate blood flow to the heart.^23^ The decreased R wave amplitude with elevation of ST segment might be due to myocardial necrosis induced by iso-proterenol. The ST segment elevation reflects the electrical potential difference in the boundary between ischemic and non-ischemic zones, whereas R wave amplitude decretion might be associated with onset of myocardial edema.^23^^,^^24^^,^^30^^,^^31^

Isoproterenol increases permeability of cardiac muscle cells leading to release of cytosolic enzymes AST, LDH and CK-MB into the blood stream.^32^ Three CK iso-enzymes including CK-MM, CK-BB and CK-MB are distributed in many tissues of the body. The CK-MB is present in a relatively high concentration in the myocardium and its activity is a useful index for diagnosis of myocardial infarction.^33^ Increase in MDA contents, the oxidative stress marker, and decrease in antioxidant enzymes (SOD and catalase) activity produced by isoproterenol may be due to increased reactive oxygen species (ROS) generation.^7^ The MDA is a reliable and common biomarker for assessing lipid peroxidation. Lipid peroxidation is a well-established mechanism of cellular injury and is used as an index of oxidative stress in cells and tissues.^34^ Histopathological changes in the heart tissue confirm the above-mentioned mechanisms used by isoproterenol to produce acute myocardial infarction. Other researchers reported similar histopathological changes in the heart tissue by isoproterenol.^23^^,^^30^^,^^31^^,^^35^ However, ROS generation plays a central role in isoproterenol-induced acute myocardial infarction.^7^

In the present study, histidine prevented isoproterenol-induced changes in heart weight, ECG patterns, serum and cardiac markers of heart injury and histopathology of the heart tissue. Although, there are not any reports showing the effects of histidine on isoproterenol-induced myocardial infarction, some evidences suggest a cardioprotective effect for histidine. In doxorubicin-induced cardiotoxicity, chronic treatment with histidine (40 mg kg^-1^, 28 days) restored ST segment elevation and QT interval prolongation, improved heart tissue hemorrhages, edema and leukocyte infiltration and prevented serum activities of LDH, CK and heart tissue MDA levelelevation.^13^ It has also been shown that histidine reduces myocardial mitochondrial damage resulted from cerebral ischemia in rats.^36^ In addition, histidine prevented post-ischemic reperfusion injury in isolated hearts by inhibiting ROS generation and preserving high-energy phosphate.^37^ In this context, histidine through scavenging of singlet oxygen, improved left ventricular developed pressure, first derivative of left ventricular pressure, heart rate and coronary flow in ischemic rat hearts.^38^

Our results showed vitamin C inhibited isoproterenol-induced ECG, biochemical and histopathological changes. Some researchers have suggested cardioprotective effects of vitamin C in isoproterenol-induced acute myocardial infarction. It has been reported that oral administration of vitamin C (80 mg kg^-1^, six days) prevents isoproterenol (150 mg kg^-1^, two days) induced changes in lipid peroxidation, serum marker enzymes (LDH, CPK, GOT and GPT) and endogenous antioxidant enzymes (SOD, GPX, GST, GSH and catalase) activities.^19^ Moreover, oral administration of a single dose (250 mg kg^-1^) and repeated doses (150 mg kg^-1^, seven days) of vitamin C before isoproterenol, reduced the extent of myocardial damage, down-regulated iNOS and improved autonomic balancing of the heart.^20^^,^^39^

Propranolol prevented the increased heart weight, heart rate, ST segment elevation, serum activities of AST, LDH and CK-MB and heart tissue level of MDA induced by isoproterenol. It also inhibited the decrease of RR interval and heart tissue antioxidant enzymes, SOD and catalase. Propranolol is a β-blocker and β-blockers exhibit cardioprotective effects in experimental myocardial infracted hearts.^7^ Calcium leakage, hemodynamic changes and elevation of heart tissue MDA induced by isoproterenol were inhibited by propranolol.^40^ The increased serum activities of LDH and CK-MB and decreased heart tissue activities of SOD and catalase induced by isoproterenol were inhibited by oral administration of 10 mg kg^-1 ^propranolol for 30 days.^8^ In this context, our study results revealed an antioxidant-based mechanism for propranolol because it significantly improved oxidative and anti-oxidative imbalance in the heart tissue.

In this study, histidine and vitamin C co-administration produced synergistic effects on the inhibition of iso-proterenol-induced changes in serum activities of AST, LDH and CK-MB and heart tissue content of MDA, SOD and catalase activities and histopathological outcomes when compared with alone use of them. These results were similar to propranolol. In this context, synergistic effect was reported between ascorbic acid and ferulic acid in reducing isoproterenol-induced myocardial infarction.^19^ Histidine and n-acetylcysteine co-administration produced synergistic effects in attenuating doxorubicin-induced changes in heart rate, ST segment, serum creatine phosphokinase (CPK) activity, cardiac tissue MDA content, hemorrhages and leukocyte infiltration.^13^ Moreover, oral administration of histidine and vitamin C reduced cyclophosphamide-induced changes in urinary bladder histology and prevented the subsequent elevation of plasma MDA content in rats.^12^ In some studies, propranolol has been used to investigate new mechanisms of β-blocker action in acute myocardial infarction induced by isoproterenol.^41^^,^^42^ In recent years, it has also been used to clarify and compare the mechanism of action of chemical agents such as natural antioxidants in isoproterenol-induced myocardial infarction.^8^^,^^40^^,^^43^ Supplementation with multiple chemical agents (two antioxidants or an antioxidant with a β-blocker in our study) is essential because several mechanisms are involved in isoproterenol-induced acute myocardial infarction.^7^

In conclusion, the results of the present study showed that isoproterenol elevates ST segment, increases heart weight, heart rate, serum activities of AST, LDH and CK-MB and heart tissue content of MDA. It decreased RR interval, R wave amplitude and cardiac tissue activities of SOD and catalase. Alone treatment with histidine and vitamin C prevented isoproterenol-induced changes in ECG pattern, serum cardiac injury biomarkers and oxidant and anti-oxidant system of the heart tissue. Combined administration of histidine with vitamin C produced a synergistic inhibitory effect on isoproterenol-induced changes in serum cardiac injury markers and oxidant and antioxidant systems of the heart that were similar to propranolol.
